# Parental Attitudes toward Artificial Intelligence-Driven Precision Medicine Technologies in Pediatric Healthcare

**DOI:** 10.3390/children7090145

**Published:** 2020-09-20

**Authors:** Bryan A. Sisk, Alison L. Antes, Sara Burrous, James M. DuBois

**Affiliations:** 1 Department of Pediatrics, Division of Hematology/Oncology, Washington University School of Medicine, St. Louis, MO 63110, USA; 2 Department of Medicine, Washington University School of Medicine, St. Louis, MO 63110, USA; aantes@wustl.edu (A.L.A.); duboisjm@wustl.edu (J.M.D.); 3 Brown School, Washington University, St. Louis, MO 63130, USA; sburrous@wustl.edu

**Keywords:** pediatrics, personalized medicine, ethics, biomedical technology, child health, artificial intelligence, machine learning, precision medicine

## Abstract

Precision medicine relies upon artificial intelligence (AI)-driven technologies that raise ethical and practical concerns. In this study, we developed and validated a measure of parental openness and concerns with AI-driven technologies in their child’s healthcare. In this cross-sectional survey, we enrolled parents of children <18 years in 2 rounds for exploratory (*n* = 418) and confirmatory (*n* = 386) factor analysis. We developed a 12-item measure of parental openness to AI-driven technologies, and a 33-item measure identifying concerns that parents found important when considering these technologies. We also evaluated associations between openness and attitudes, beliefs, personality traits, and demographics. Parents (*N* = 804) reported mean openness to AI-driven technologies of M = 3.4/5, SD = 0.9. We identified seven concerns that parents considered important when evaluating these technologies: quality/accuracy, privacy, shared decision making, convenience, cost, human element of care, and social justice. In multivariable linear regression, parental openness was positively associated with quality (beta = 0.23), convenience (beta = 0.16), and cost (beta = 0.11), as well as faith in technology (beta = 0.23) and trust in health information systems (beta = 0.12). Parental openness was negatively associated with the perceived importance of shared decision making (beta = −0.16) and being female (beta = −0.12). Developers might support parental openness by addressing these concerns during the development and implementation of novel AI-driven technologies.

## 1. Introduction

Precision medicine offers the hope of transforming healthcare by developing more accurate and effective strategies to diagnose, prevent, and treat diseases by considering an individual’s genetic, environmental, and lifestyle characteristics [1,2]. To fulfill these hopes, precision medicine interventions require access to vast amounts of personal-level data and patient information, as well as artificial intelligence (AI) algorithms capable of analyzing these large datasets [3]. Recent advances in AI technologies, such as machine learning and deep learning, have further increased enthusiasm about the prospects of precision medicine transforming healthcare [4,5,6,7,8,9]. In pediatrics, AI-driven technologies are beginning to enter clinical studies in the management of diabetes mellitus [10], asthma [11], and cancer [12].

However, these evolving AI-driven technologies also raise ethical and practical concerns [3], especially in pediatrics. For example, large genetic, environmental, and lifestyle datasets from diverse populations of children are necessary for precision medicine to advance. Some parents might worry about the privacy of their child’s data or the transparency of how these data will be used. Other parents might worry about the impact of these interventions on the clinician–family relationship. Some have argued, for example, that an influx of AI-driven technologies will further increase the clinician’s reliance on computers and electronic medical records at the expense of genuine human engagement [13].

For the field of pediatrics to realize the benefits of precision medicine, researchers and developers must have access to large amounts of diverse data and children must utilize these technologies. Parental openness and support of these technologies will likely expedite this development. As such, it is imperative to understand the ethical and practical concerns that affect parental openness to these AI-driven technologies. In this study, we developed and validated a novel measure of parental openness to the use of AI-driven healthcare technologies in their child’s medical care, and we identified factors that parents considered important in evaluating these technologies.

## 2. Materials and Methods

We report this study in keeping with the Checklist for Reporting Results of Internet E-Surveys (CHERRIES) [14]. (Appendix A) This project proceeded in several stages: expert interviews; literature review; item writing; cognitive interviewing and item revision; and two rounds of survey distribution for exploratory and confirmatory factor analysis.

### 2.1. Item Writing

To establish the content to be presented in the original measure, we conducted seven professional informant interviews with bioinformaticians, bioethicists, health lawyers, and physicians. We asked experts to provide their definition of AI, how they would explain AI to non-experts, to highlight clinical examples of AI (both present and future), and to enumerate potential concerns and benefits of AI in healthcare. We interviewed these leading experts to identify key ethical and practical concerns and to ensure our interpretation of the literature was accurate. We cross-referenced their responses with a literature review of more than 300 articles. For this literature review, we consulted with a medical librarian and used Boolean logic to search PubMed, Scopus, and Embase for articles related to “artificial intelligence”, “machine learning”, “big data”, and “health care.” We subsequently searched for clinical applications, ethical concerns, and patient perspectives within these broad searches. We did not restrict searches to pediatrics in order to understand the broad landscape of the AI field of study. After screening, we identified more than 300 articles related to practical and ethical aspects of AI. Additionally, we identified review articles and commentaries that highlighted major ethical issues in the use of AI in healthcare [3,15,16,17,18,19]. From this review, we developed a framework of nine factors that might influence openness to AI-driven healthcare interventions in pediatrics: privacy, transparency, human element of care, social justice, societal cost, personal cost, quality/accuracy, access to care, and access to knowledge. We hypothesized that these factors represented latent constructs that captured the breadth of parental concerns, and we wrote items that targeted each of these constructs.

We next developed an original measure, Attitudes toward Artificial Intelligence in Pediatric Healthcare (AAIH-P), to assess openness to the use of AI-driven technologies in their child’s healthcare and to identify concerns that parents considered important when considering engagement with these devices. This new measure consisted of a general openness scale and a scale that measured the importance of various concerns in the parent’s consideration of these technologies. We developed the openness scale with the intention of understanding parental openness to a variety of AI-driven applications. We created the concerns scale with the goal of determining whether our hypothesized factors represented underlying latent constructs, and whether these concerns were associated with the openness scale.

Of note, we opted to avoid using the terms ‘artificial intelligence’ or ‘machine learning’ in these measures because of misconceptions and complexity associated with these terms, based on feedback from informant interviews with professionals. We also asked these professionals how they would describe AI or machine learning to lay audiences. These descriptions focused on the need for large amounts of data, the ability to make comparisons to many other people similar to the patient, and the unique clinical functions these AI-driven technologies might offer. Due to this, we opted to describe characteristics of the technologies without labeling them as ‘artificial intelligence’ or ‘machine learning’. During cognitive interviews, parents preferred the terms ‘computer programs’ and ‘devices’, so we opted for this phrasing.

The general openness scale asked participants, “How open are you to allowing computer programs to do the following things?” Participants were asked to read 12 items and select their level of openness on a five-point Likert scale ranging from ‘not at all open’ (1) to ‘extremely open’ (5). These 12 items represented four different AI-driven healthcare functions, including diagnosis (e.g., “Determine if your child broke a bone”), risk prediction (e.g., “Predict your child’s risk of developing depression in the future”), treatment selection (e.g., “Decide on the best treatment for your child’s diabetes”), and medical guidance (e.g., “Give you advice on how to prevent your child’s asthma attacks”) (Appendix B). We included interventions that we believed represented a wide range of emotional intensity for the respondents. This determination of emotional intensity was based on clinical expertise of one author (BAS) in treating children with minor complaints (e.g., ear infections) and diagnosing children with serious, life-threatening diagnoses (e.g., cancer). To support face and content validity, we asked parents during cognitive interviews about their emotional reactions to the range of diagnoses, and whether they were familiar with these diagnoses.

The second component of this measure aimed to identify concerns that parents found important when considering the use of AI-driven healthcare interventions in their child’s medical care. Participants were asked, “When you think of using these new devices, how important are the following details to you?” Each of the items described a potential aspect of AI-driven healthcare interventions that might affect openness. For example, “Whether these devices are better than your child’s doctor at figuring out why your child is sick.” Participants selected how important these items were on a five-point Likert scale, ranging from ‘not important’ (1) to ‘extremely important’ (5). We drafted 57 initial survey items that targeted 8 hypothetical constructs (collapsing personal cost and societal cost into a single construct). We wrote 4 to 10 items per hypothetical factor, using both positive and negative valence to increase variance in responses. 

After drafting this measure, we performed 11 cognitive interviews to ensure face validity, item clarity, and content validity. We subsequently revised the measure before distributing [20]. For these cognitive interviews, we recruited mothers and fathers whose children were younger than 18 years old with a broad range of racial/ethnic, professional, socioeconomic, and educational backgrounds. To identify participants for these cognitive interviews, members of the research team approached acquaintances who could provide diverse perspectives. We prioritized diversity in education level and racial background. In these interviews, we reviewed surveys item-by-item for readability and clarity. For select items, we asked interviewees to rephrase the question in their own words, to ensure understandability. We also asked participants if they recommended any changes to information, scenarios, wording, or content. For any problematic questions or terminology, we asked what made the question hard to answer and what might help them to answer the question. We also learned about parents’ emotional reactions to the range of diagnoses, and whether they were familiar with these diagnoses. Lastly, we asked for general comments about the measure and scope of the items. Most commonly, parents raised concerns with specific words of phrasings that were confusing. Parents did not raise concerns about content of scenarios. We also assessed for reading ease and grade level, achieving Flesch–Kincaid Grade Level of 7.6.

### 2.2. Survey Participants

We employed criterion-based sampling to recruit participants using Amazon’s Mechanical Turk (MTurk), a web-based service that matches ‘requesters’ with ‘workers’ to complete various ‘human intelligence tasks’ (HITs). Participants were required to be parents whose children were all 18 years or younger. Past studies have demonstrated that MTurk is a cost-effective and reliable means of recruiting nationally representative samples of parents for online surveys [21]. We applied specific MTurk qualifications, stipulating that participants have a 98% approval rating on at least 100 prior HITs to qualify for this survey. Additionally, we applied custom qualifications to prevent multiple entries from the same individual. Eligible participants completed the survey through a web-link to Qualtrics survey software. Participants received $3.65 for completing this 30-min task. All subjects gave their informed consent for inclusion before they participated in the study. The study was conducted in accordance with the Declaration of Helsinki, and the protocol was approved by the Ethics Committee of Washington University School of Medicine (202004198). Participants reviewed an exempt information sheet at the beginning of the survey indicating the purpose of the study, the identity of the researchers, and the anticipated time commitment. Data were stored on encrypted servers.

We administered this survey in two rounds to perform exploratory factor analysis (EFA) followed by confirmatory factor analysis (CFA). Round 1 collected 449 responses, and round 2 collected 451 responses. Surveys were opened on 23 April 2020 and 11 May 2020. For the AAIH-P measure, each screen of the questionnaire contained 6 to 8 items. In total, the full battery of surveys contained 14 screens of questions. We utilized forced entry, so no questionnaires were incomplete. Respondents were not able to review or change answers from prior screens. Completion rates were 449/508 (88%) and 451/498 (91%) for rounds 1 and 2, respectively. 

For quality control, we excluded participants who completed the survey in less than 7 min, anticipating that each question should require 4–5 s to respond meaningfully. For this reason, we excluded 20 participants (4%) in the first round and 54 (11%) in the second round. Additionally, we excluded 11 participants from each round who provided ages of children that were greater than 18 years. After exclusions, round 1 included 418 participants and round 2 included 386 participants, for a total of 804 participants in the entire study.

### 2.3. Validity and Psychometric Properties of AAIH-P Measure

To assess the validity of the AAIH-P openness measure, we assessed for internal reliability and convergent validity with other validated scales. To assess the validity of the AAIH-P concerns measure, we performed exploratory and confirmatory factor analysis.

#### 2.3.1. AAIH-P Openness Scale

For the 12-item AAIH-P openness scale, we computed the mean response to items to create a composite score that ranged from 1 to 5. To test for internal reliability, we calculated Cronbach’s alpha for the full 12-item scale (α = 0.92). We also calculated mean responses and Cronbach’s alpha for the items related to the four different AI-driven healthcare functions: diagnosis (α = 0.84), risk prediction (α = 0.87), treatment selection (α = 0.90), and medical guidance (α = 0.84).

#### 2.3.2. Exploratory Factor Analysis

We performed EFA with Promax rotation to assess the factor structure of the AAIH-P concerns scale. We examined the scree plot of eigenvalues and considered the amount of variance explained by each additional factor in order to determine the total number of factors to include. We also considered the number and magnitude of loadings on factors across potential factor solutions [22]. Factorability of the items was confirmed by a significant Bartlett’s test of sphericity, v2 (1596) = 12,393.25, *p* < 0.001, and Kaiser–Meyer–Olkin value of greater than 0.6 (KMO = 0.92).

Results of the EFA indicated a seven-factor solution that accounted for 54% of variance. We dropped two items that loaded poorly on these seven factors, and we dropped 16 items because of significant cross-loading on other factors (>0.3). To decrease survey burden, we dropped eight items with content that was captured in other similar items. After dropping these items, we repeated EFA with the 34 remaining items, finding that a seven-factor solution explained 60% of variance (see Table 1 for definitions of factors).

#### 2.3.3. Confirmatory Factor Analysis

We then distributed the battery of surveys containing the revised 34-item AAIH-P concerns scale and performed CFA using maximum likelihood estimation. We allowed all factors to correlate with each other. We removed one item with poor fit. There was acceptable fit between the finalized model and the observed data: χ^2^ *p* < 0.001, CFI = 0.91, RMR = 0.069, RMSEA = 0.053.

#### 2.3.4. Measures of Sociodemographic Attributes, Attitudes, and Personality Traits

To assess for convergent validity of the AAIH-P openness scale, we also administered measures of sociodemographic attributes, attitudes, and personality traits and examined associations with openness on AAIH-P. The Ten Item Personality Inventory (TIPI) is a 10-item measure used to assess personality constructs: openness to new experiences, conscientiousness, extraversion, agreeableness, and emotional stability [47]. We anticipated a positive relationship between AAIH-P openness scale and openness as a personality trait. We assessed participants’ trust in health information systems using Platt’s scale. This 20-item scale includes four subscales: Fidelity, Competency, Trust, and Integrity [48]. We also included a measure of faith and trust in general technology [49]. We hypothesized a positive correlation between measures of trust and openness scores on AAIH-P.

We asked participants which political party they most align with: Democrat, Republican, Independent, or other. For respondents who chose independent or other, we asked whether they leaned democrat or leaned republican. For analysis, we clustered democrat with lean democrat and republican with lean republican. We hypothesized that participants who align with the Republican Party would be more conservative and might have lower openness on AAIH-P.

Lastly, we included the Positive and Negative Affect Scale (PANAS) to assess for the impact of the participant’s current affect on responses. This survey was administered during the early phases of the COVID−19 pandemic, and we hypothesized that negative affect generated by the societal challenges might negatively correlate with openness.

### 2.4. Statistical Analyses

We examined correlations between openness on AAIH-P and the other scales and demographic variables using Pearson (continuous variables) or Spearman (ordinal variables) correlations. To further explore the relationships between these variables and openness, we performed multiple linear regression with stepwise entry, excluding non-significant variables (*p* < 0.05). In the original model, we included the scores of all AAIH-P concerns subscales, race, and all other variables that demonstrated a significant correlation (*p* < 0.05) in bivariable correlations with openness. Given the number of included variables and potential for intercorrelation, we performed iterative calculations of variance inflation factor (VIF) to assess for multicollinearity. All VIFs were <5 and most were <2, indicating low likelihood of multicollinearity. We performed analyses in IBM SPSS statistical package v26.0.0.0, using Amos Structural Equation Modeling plug-in for CFA.

## 3. Results

### 3.1. Participant Characteristics

The majority of participants were female (59%, 470/804), and the mean (M) age was M = 38.9, standard deviation (SD) = 8 years. Most participants were non-Hispanic (93%, 751/804) and White (86%, 689/804). Additionally, 10% (77/804) were Black, 6% (44/804) were Asian, and 3% (20/804) were American Indian or Alaska Native. Most participants had completed a Bachelor’s or graduate degree (59%, 481/804). Participants had a median of 2 children, ranging from 1 to 8. (Table 2).

### 3.2. Openness to AI-Driven Healthcare Interventions and Parental Concerns

Parents had a mean openness score on the AAIH-P of M = 3.4 (on a five-point scale), SD = 0.9. Openness to the four different AI-driven healthcare functions were as follows: diagnosis M = 3.8, SD = 1.1; risk prediction M = 3.1, SD = 1.2; treatment selection M = 3.2, SD = 1.2; medical guidance M = 3.6, SD = 1.0. EFA and CFA identified seven concerns that parents considered important when evaluating the use of AI-driven healthcare interventions in their child’s medical care. (Table 1) Quality (M = 4.3, SD = 0.8), privacy (M = 4.2, SD = 0.8), and shared decision making (M = 4.1, SD = 0.8) received the highest mean importance ratings. Convenience (M = 3.8, SD = 0.8), cost (M = 3.7, SD = 1.0), human element (M = 3.5, SD = 0.9), and social justice (M = 3.5, SD = 1.0) were rated lower.

### 3.3. Relationships between Openness on AAIH-P and Parent Concerns and Characteristics

Of the seven concerns identified in factor analysis, four concerns correlated significantly and positively with openness: social justice, quality, cost, and convenience. Measures of trust also correlated positively with openness: all Platt subscales, Faith in Technology, and Trust in Technology. Alignment with the Democratic Party or leaning toward the Democratic Party correlated positively with openness. Three personality traits correlated positively with AAIH-P openness: extroversion, openness to change, and emotional stability. Positive affect also correlated positively with openness. Of demographic variables, being female negatively correlated with openness (Table 3).

In multivariable linear regression, nine variables showed a significant association with parental openness to AI-driven interventions in their child’s healthcare. Of concerns, quality (β =0.23, 95% Confidence Interval (CI) 0.16 to 0.31), convenience (β = 0.16, 95% CI 0.09 to 0.23), and cost (β = 0.11, 95% CI 0.04 to 0.17) were positively associated with openness. Shared decision making was negatively associated with openness (β = −0.16, 95% CI −0.23 to −0.10). Two measures of trust were positively associated with openness: Faith in Technology (β = 0.23, 95% CI 0.17 to 0.29) and Platt Trust subscale (β = 0.12, 95% CI 0.06 to 0.18). Alignment with the Democratic Party (β = 0.09, 95% CI 0.03 to 0.15) and extroversion (β = 0.04, 95% CI 0.01 to 0.07) had small positive associations with openness, and being female had a negative association (β = −0.12, 95% CI −0.18 to −0.06) (Table 4).

Although parents reported that privacy was highly important to them, privacy scores were not associated with openness scores. To further explore the role of privacy, we examined an exploratory multivariable model with openness as the dependent variable and the seven factors as predictor variables. In this model, privacy had a small, negative association with AAIH-P openness (β = −0.08, 95% CI −0.155 to −0.01). We then explored the correlations between privacy and measures of trust, finding that privacy was negatively correlated with the trust subscale of the Platt measure (r = −0.10, *p* = 0.03) and the trust in technology scale (r = −0.11, *p* = 0.002).

## 4. Discussion

In this study, we developed the first measure of openness to AI-driven healthcare interventions in pediatrics. Our study provided evidence of face, content, and construct validity for this measure of openness, as well as factorial validity for the measure of concerns. In administration to 804 participants, we found that parents were moderately open to the use of AI-driven healthcare interventions in their child’s medical care. We also identified seven categories of concerns that parents found important in considering the use of these technologies. These results reinforce prior findings from the Future Advocacy initiative, in which researchers interviewed 71 professionals with experience in AI and 16 members of the public to identify potential ethical, social, and political implications of AI in healthcare. The authors identified issues related to the impact of AI on human relationships in health and care, storage and sharing of medical data, algorithmic transparency/explainability, health-related disparities, and trustworthiness or reliability of these interventions, among others [3]. Others have similarly highlighted concerns about privacy, transparency, and the role of the physician as AI-driven interventions become incorporated into medical practice [50,51]. However, ours is the first study to verify the importance of these issues to parents in a large, quantitative survey.

On average, parents considered each of these concerns to be important or very important. In a multivariable model, quality of the intervention, convenience, and cost were positively associated with openness. Given this positive association, it is possible that parents who are more open to these technologies believe that AI-driven technologies will improve the cost, quality, and convenience of pediatric healthcare. Conversely, parents who rated shared decision making highly were less likely to be open. This finding suggests that some parents worry these technologies might decrease their role in making decisions on behalf of their child. Several studies have identified “making informed decisions” as central to “good parenting” beliefs for parents of seriously ill children [52,53,54,55,56]. By ensuring that new technologies engage parents in the decision-making process, researchers and developers might enhance parental openness to using these technologies in their child’s care. Beyond these factors, parental reports of faith in technology and trust in public health information sharing were also associated with openness.

Interestingly, the privacy subscale was not associated with parental openness to these technologies, despite parents rating privacy items as highly important. In exploratory analyses, we found that privacy scores correlated negatively with trust scales. Furthermore, we found that privacy scores were significantly associated with openness when we excluded trust scores from the multivariable model. These findings suggest that privacy and trust scales are measuring similar underlying constructs. If parents have lower trust, then they will tend to be more concerned about privacy. As such, privacy concerns might affect openness to the extent that they change the parent’s level of trust. However, these findings were exploratory, and the role of privacy concerns should be more fully explored in future studies.

These findings provide important insights into parental openness to AI-driven healthcare for their children, and they indicate several characteristics of parents and interventions that might affect this openness. AI researchers and developers should consider these variables when designing and implementing novel interventions. By addressing these ethical and practical concerns early in the development process, parents might be more likely to engage with precision medicine research and applications. Without this engagement, children might be left behind as precision medicine advances in other disciplines.

These results, however, should be interpreted in light of limitations. First, parents in this sample were predominantly White with college education. Given the concern that precision medicine could exacerbate racial and socio-economic disparities [57,58], future studies should engage with medically underserved communities to ensure adequate representation of their values and beliefs. Additionally, only 3% of parents reported that their children had been hospitalized in the last 12 months. Future studies should engage parents of children with serious illnesses to explore the impact of children’s health status on parental openness. Also, this survey was administered through MTurk to participants who are likely comfortable with technology. As such, the openness of this sample could be skewed to more openness to novel technologies, potentially limiting the generalizability of these results. This sample was also skewed toward higher education and income. However, our sample was large, we did have adequate numbers of people in the lower education and income categories to detect modest differences. There were no significant differences or trends in the data. We believe this reduces the negative effect of this demographic skew on our conclusions. Lastly, we only identified concerns that were in our original framework. While our cognitive interviews provided important insights, our study could have been strengthened by more qualitative work with parents to potentially identify additional of concerns related to AI in their child’s healthcare. Future explanatory mixed-methods studies should explore whether parents have additional concerns not represented in this measure.

AI-driven precision medicine could transform pediatric care in the future. However, development of these technologies requires analysis of large amounts of data from many children. Thus, parental openness to the use of these AI-driven technologies in their child’s care is paramount. In this study, we developed and validated a novel measure of parental openness to the use of AI-driven precision medicine interventions in their child’s care. We also found that parental openness is associated with perceptions of the intervention’s impact on cost, quality, convenience, and shared decision making, as well as trust in information sharing systems, faith in technology, and the parents’ gender. These findings can inform strategies for intervention development and engagement with parents in the future.

## Figures and Tables

**Table 1:** Parental concerns with AI-driven healthcare technologies

Parental Concerns	Description
Social justice	Concerns about how these new technologies might affect the distribution of the benefits and burdens related to AI use in healthcare [3,9,16,17,19,23,24,25,26,27,28,29].
Human element of care	Concerns about the effect of these technologies on the interaction or relationship between clinicians and patients/families [3,30].
Cost	Concerns about whether these technologies will affect individual or societal costs [31,32].
Convenience	Concerns about the ease with which an individual can access and utilize these technologies [33,34,35,36,37].
Shared decision making	Concerns about parental involvement and authority in deciding whether and how these technologies are utilized in their child’s care.
Privacy	Concerns about loss of control over the child’s personal information, who has access to this information, and how this information might be used [3,17,36,38,39,40,41,42].
Quality and accuracy	Concerns about the effectiveness and fidelity of these technologies [43,44,45,46].

**Table 2:** Participant characteristics

Participant Characteristics (*N* = 804)	*n* (%), Except Where Specified
Age of parent, Mean (SD)	38.9 years (8.0)
Female sex	470 (59%)
Race *	
White	689 (86%)
Black or African American	77 (10%)
Asian	44 (6%)
American Indian or Alaska Native	20 (3%)
Native Hawaiian or Pacific Islander	2 (<1%)
Hispanic ethnicity	53 (7%)
Employment status	
Full-time	560 (70%)
Part-time (not a full-time student)	48 (6%)
Full-time student	2 (<1%)
Self-employed	61 (8%)
Caregiver or homemaker	93 (11%)
Other	40 (5%)
Household income	
Less than 23,000	42 (5%)
23,001–45,000	150 (19%)
45,001–75,000	263 (32%)
75,001–112,000	208 (26%)
Greater than 112,001	138 (17%)
Level of education	
Some high school	5 (<1%)
High school graduate	61 (8%)
Some college	141 (18%)
Associate’s degree	116 (14%)
Bachelor’s degree	343 (42%)
Master’s or doctoral degree	138 (17%)
Place of residence	
Urban	176 (22%)
Suburban	460 (57%)
Rural	168 (21%)
Health insurance status	
Private	595 (74%)
Medicaid	116 (14%)
Medicare/Medicare Advantage	47 (6%)
No health insurance	30 (4%)
Other	16 (2%)
Number of children, median (IQR)	2 (1 to 2)
Child visited doctor in past 12 months	709 (88%)
Number of doctor visits, median (IQR)	8 (6 to 10)
Child hospitalized in past 12 months	27 (3%)
Number of hospitalizations, median (IQR)	1 (1 to 2)

* Race responses were not mutually exclusive. Missing data due to selecting ‘prefer not to answer’: Sex (1), Race (5), Household Income (3), Level of Education (1), Political Alignment (1). SD = standard deviation. IQR = interquartile range.

**Table 3:** Bivariable correlations with openness to AI-driven interventions in pediatrics

Variable (Cronbach’s Alpha)	Correlation (*p* Value)
*Concerns*	
**Social justice (0.86)**	**0.22 (<0.001)**
Human element (0.72)	0.02 (0.49)
**Cost (0.81)**	**0.25 (0.001)**
**Convenience (0.69)**	**0.31 (0.001)**
Shared decision making (0.72)	−0.01 (0.84)
Privacy (0.87)	−0.03 (0.45)
**Quality (0.71)**	**0.34 (<0.001)**
*Attitudes, Beliefs, and Personality Scales*	
**Platt fidelity (0.71)**	**0.11 (0.002)**
**Platt competency (0.78)**	**0.21 (<0.001)**
**Platt trust (0.95)**	**0.23 (<0.001)**
**Platt integrity (0.85)**	**0.20 (<0.001)**
**TIPI extroversion (0.80)**	**0.13 (<0.001)**
**TIPI openness (0.59)**	**0.13 (<0.001)**
TIPI agreeableness (0.46)	0.06 (0.10)
TIPI conscientiousness (0.73)	0.06 (0.08)
**TIPI emotional stability (0.83)**	**0.10 (0.01)**
**Faith in technology (0.87)**	**0.36 (<0.001)**
**Trust in technology (0.91)**	**0.28 (<0.001)**
**PANAS–Positive affect (0.91)**	**0.17 (<0.001)**
PANAS–Negative affect (0.91)	0.01 (0.81)
**Democrat/lean Democrat political alignment**	**0.09 (0.015)**
**Demographic Variables**	
**Female sex**	**−0.13 (<0.001)**
Age	−0.04 (0.294)
Race (White vs Person of Color) *	0.04 (0.238)
Ethnicity (Hispanic vs non-Hispanic)	−0.04 (0.248)
Worked in healthcare field	−0.01 (0.780)
Income	0.03 (0.357)
Highest level of education	0.03 (0.468)
Number of children	−0.05 (0.149)
Number of children’s doctor visits **	−0.05 (0.773)
Child hospitalization	<0.01 (0.889)

We used Spearman correlation for concerns and all demographic variables except age. Bolding indicates *p* < 0.05. We used Pearson correlation for all remaining correlations. * Participants who selected any race category other than White or in addition to White were considered “Persons of Color” in this analysis. ** Excluded 98 responses of parents whose children had no doctor’s visits. TIPI = Ten Item Personality Inventory. PANAS = Positive and Negative Affect Scale.

**Table 4:** Multivariable model of variables associated with openness to AI-driven interventions in pediatric healthcare

Variable	Standardized β Coefficient (95% CI)	*p* Value
*Concerns*		
Quality	0.23 (0.16 to 0.31)	<0.001
Convenience	0.16 (0.09 to 0.23)	<0.001
Cost	0.11 (0.04 to 0.17)	0.001
Shared decision making	−0.16 (−0.23 to −0.10)	<0.001
*Attitudes, Beliefs, and Personality Scales*		
Faith in technology	0.23 (0.17 to 0.29)	<0.001
Platt trust	0.12 (0.06 to 0.18)	<0.001
Democrat/lean Democrat	0.09 (0.03 to 0.15)	0.002
TIPI extroversion	0.04 (0.01 to 0.07)	0.015
*Demographic Variables*		
Female sex	−0.12 (−0.18 to −0.06)	<0.001

Multiple linear regression with stepwise entry. We initially included scores of all concern subscales, all other variables with significant correlation to openness and race.

**Figure A1:**
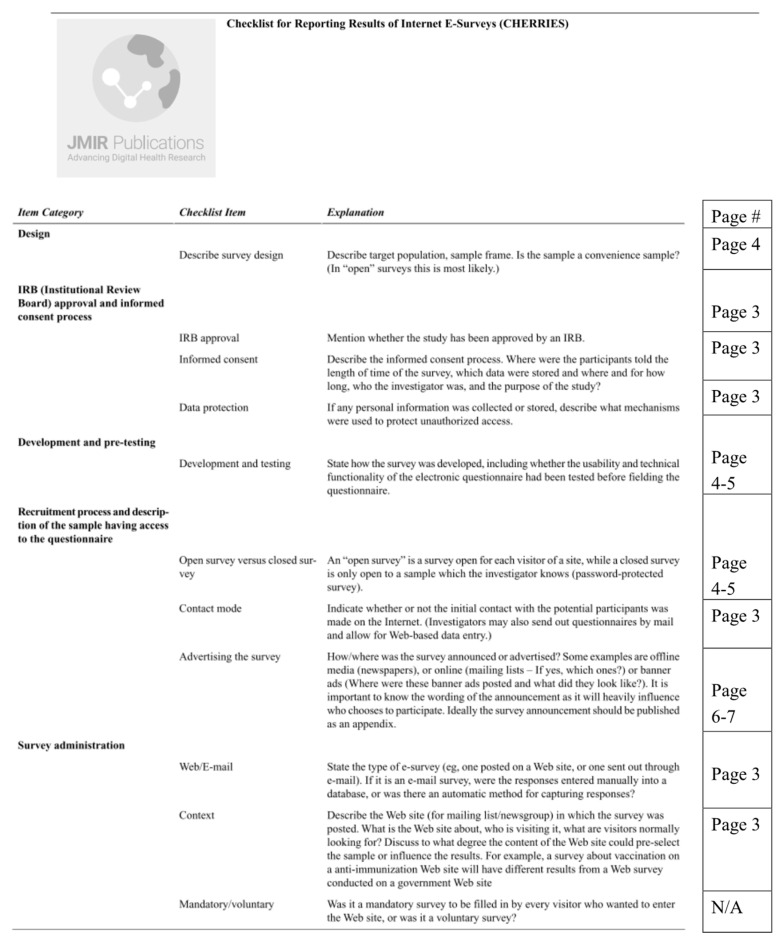

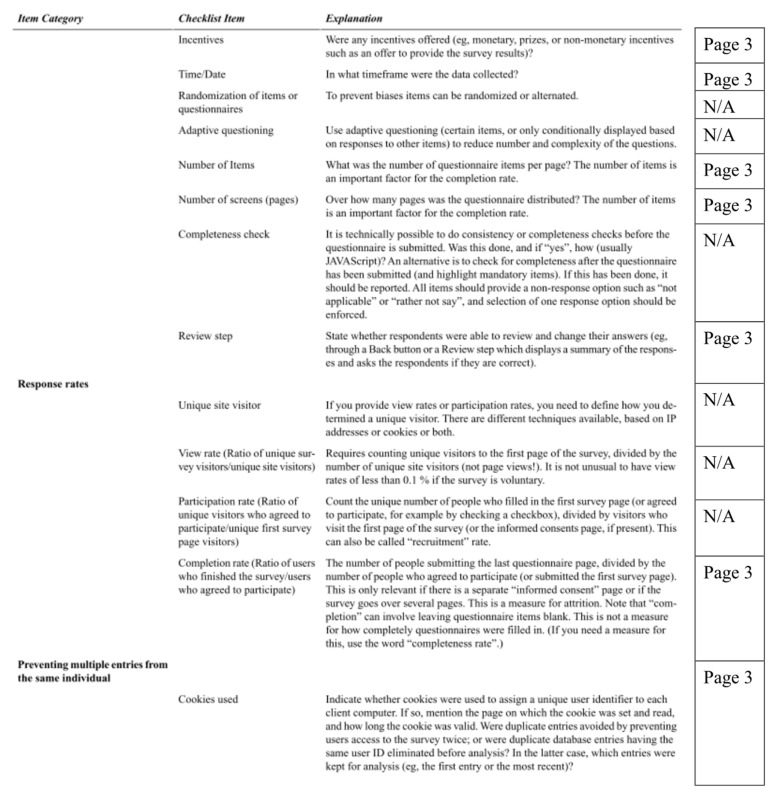

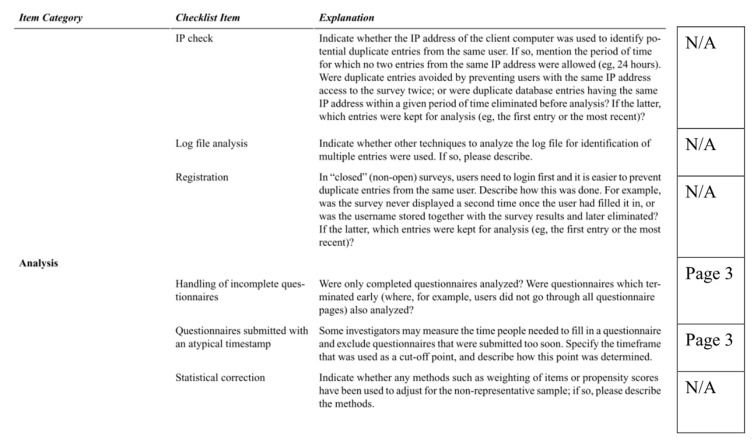
Cherries Checklist.

## Appendix B. Full AAIH-P Measure with Factors Labeled

**Introduction:** Overview: In this survey, we describe healthcare devices that use advanced computer programs. Some of the devices already exist. Others might soon exist. These devices are becoming common. We want to know your views about them. There are no right or wrong answers.

**How Open Are You to Allowing Computer Programs to Do the Following Things?**	**Please Rate Your Response for Each Statement**				
(1) Determine if your child has an ear infection.	1	2	3	4	5
(2) Determine if your child broke a bone.	1	2	3	4	5
(3) Determine if your child has cancer.	1	2	3	4	5
(4) Predict your child’s risk of developing obesity in the future.	1	2	3	4	5
(5) Predict your child’s risk of developing depression in the future.	1	2	3	4	5
(6) Predict your child’s chances of developing a disease that cannot be cured.	1	2	3	4	5
(7) Decide on the best treatment for your child’s lung infection.	1	2	3	4	5
(8) Decide how often your child needs pain medication after surgery.	1	2	3	4	5
(9) Decide the best treatment for your child’s diabetes.	1	2	3	4	5
(10) Give your child feedback on how to prevent anxiety attacks.	1	2	3	4	5
(11) Recommend if you should bring your child to the hospital after an injury.	1	2	3	4	5
(12) Give you advice on how to prevent your child’s asthma attacks.	1	2	3	4	5
1—Not at all open, 2—Somewhat open, 3—Open, 4—Very open, 5—Extremely open.					

Many things are important when considering whether to use these new devices in your child’s medical care. We want to understand which things are most important to you, and which things are the least important to you. In the next set of questions, please pick the response that best matches your feelings about these devices.

**When You Think of Using These New Devices, How Important Are the Following Details to You?**	**Please Rate Your Response for Each Statement**				
(1) Whether these devices help you to weigh the risks and benefits of different treatment options for your child’s illness. *(Quality and Accuracy)*	1	2	3	4	5
(2) Whether companies that develop these devices share your child’s information with other companies. *(Privacy)*	1	2	3	4	5
(3) Whether these devices are available to low income families. *(Social Justice)*	1	2	3	4	5
(4) Whether these devices allow your child’s doctor to spend more time with you during clinic visits. *(Human Element)*	1	2	3	4	5
(5) Whether these devices reduce medical errors. *(Quality and Accuracy)*	1	2	3	4	5
(6) Whether you might save money by using these devices in your child’s medical care. *(Cost)*	1	2	3	4	5
(7) Whether these devices make it harder to have a personal relationship with your child’s doctor. *(Human Element)*	1	2	3	4	5
(8) Whether these devices provide information that is easy to understand. *(Quality and Accuracy)*	1	2	3	4	5
(9) Whether families without health insurance can access these devices. *(Social Justice)*	1	2	3	4	5
(10) Whether families in rural areas can access these devices. *(Social Justice)*	1	2	3	4	5
(11) Whether you will have to pay extra to use these devices for your child’s medical care. *(Cost)*	1	2	3	4	5
(12) Whether these devices reduce the wait time to schedule an appointment with your child’s doctor. *(Convenience)*	1	2	3	4	5
(13) Whether these devices make it easier to have a personal relationship with your child’s doctor. *(Human Element)*	1	2	3	4	5
(14) Whether your child’s doctor asks you for permission before using these devices in your child’s medical care. *(Shared Decision Making)*	1	2	3	4	5
(15) Whether the doctor asks your opinion before prescribing the treatments recommended by these devices. *(Shared Decision Making)*	1	2	3	4	5
(16) Whether these devices are accurate. *(Quality and Accuracy)*	1	2	3	4	5
(17) Whether you know how your child’s medical information will be used by the company that owns this technology. *(Privacy)*	1	2	3	4	5
(18) Whether you are better able to prevent your child from getting sick because of these devices. *(Quality and Accuracy)*	1	2	3	4	5
(19) Whether you can get your child’s medical test results more quickly because of these devices. *(Convenience)*	1	2	3	4	5
(20) Whether your insurance costs will increase to pay for these devices. *(Cost)*	1	2	3	4	5
(21) Whether these devices are widely available to everyone. *(Social Justice)*	1	2	3	4	5
(22) Whether these devices will store pictures of your child in an online database. *(Privacy)*	1	2	3	4	5
(23) Whether these devices can answer your healthcare questions. *(Quality and Accuracy)*	1	2	3	4	5
(24) Whether these devices will increase costs to the overall healthcare system. *(Cost)*	1	2	3	4	5
(25) Whether people living with disabilities can access these devices. *(Social Justice)*	1	2	3	4	5
(26) Whether computer hackers could steal your child’s medical information from these devices. *(Privacy)*	1	2	3	4	5
(27) Whether these devices are equally accurate when used for people of different races and ethnicities. *(Social Justice)*	1	2	3	4	5
(28) Whether the company collects more information than it needs about your child. *(Privacy)*	1	2	3	4	5
(29) Whether your child’s medical information is kept private. *(Privacy)*	1	2	3	4	5
(30) Whether your child’s doctor tells you if these devices are used in your child’s care. *(Shared Decision Making)*	1	2	3	4	5
(31) Whether these devices help your child get medical care with fewer doctor visits. *(Convenience)*	1	2	3	4	5
(32) Whether the doctor orders the treatments recommended by these devices without first asking your opinion. *(Shared Decision Making)*	1	2	3	4	5
(33) Whether a computer will answer most of your questions instead of people. *(Human Element)*	1	2	3	4	5
1—Not Important, 2—Somewhat Important, 3—Important, 4—Very Important, 5—Extremely Important.					

Note: Items are labeled with the subscale they are measuring (e.g. ‘human element’, ‘privacy’, et al.) for the convenience of the reader. These italicized labels should not be included when the measure is administered to parents.
